# Altitude-Driven Variations in Nutritional, Bioactive, and Mineral Profiles of Hawthorn (*Crataegus* spp.)

**DOI:** 10.3390/foods14020241

**Published:** 2025-01-14

**Authors:** Yanyan Liu, Lu Chen, Guohui Shen, Yanting Gu, Yanzhi Guo, Juan Han

**Affiliations:** 1 College of Food Science and Engineering, Qingdao Agricultural University, Qingdao 266109, China; liuyanyan202202@163.com; 2 Institute of Food and Nutrition Development, Ministry of Agriculture and Rural Affairs, Beijing 100081, China; 3 Chinese Academy of Agricultural Sciences, Beijing 100081, China

**Keywords:** hawthorn, altitude, bioactive compounds, nutritional profiles, functional food

## Abstract

Hawthorn (*Crataegus* spp.), a plant widely distributed in temperate and subtropical regions, is valued for its bioactive compounds and diverse health benefits. Known for its remarkable adaptability to various environmental conditions, hawthorn thrives across different altitudes, but these environmental factors, particularly altitude, significantly influence the accumulation of its bioactive substances. This study investigates the effects of altitude on hawthorn’s nutritional, bioactive, and mineral profiles to provide insights into its cultivation and utilization. Through comprehensive analysis of 20 nutritional indicators from high- and low-altitude samples, including essential nutrients, bioactive compounds, and trace elements, multivariate analyses such as Principal Component Analysis (PCA) and Partial Least Squares Discriminant Analysis (PLS-DA) revealed clear altitude-driven clustering. While primary nutritional components like dietary fiber, protein, and soluble solids exhibited stability across different altitudes, low-altitude samples showed higher levels of hypericin, quercetin, and rutin, likely due to favorable light and temperature conditions. Conversely, high-altitude samples were enriched in calcium, reflecting adaptations to cold stress and structural needs, while phosphorus content was reduced under cooler conditions. Potassium, iron, zinc, selenium, and strontium levels remained stable, indicating robust metabolic regulation. These findings confirm the significant role of altitude in shaping hawthorn’s bioactive and mineral profiles, providing essential guidance for altitude-specific cultivation practices and tailored processing strategies. By leveraging these insights, the functional and nutritional properties of hawthorn can be optimized, supporting its sustainable application in the food and health industries.

## 1. Introduction

Hawthorn (*Crataegus* spp.), a genus the family Rosaceae, is widely distributed across temperate and subtropical regions of the Northern Hemisphere [1,2], particularly in mountainous and hilly areas. The nutritional and functional value of hawthorn stems from its richness in bioactive compounds, including vitamin C, dietary fiber, flavonoids, and phenolic acids [3,4]. These compounds are associated with various health-promoting properties, such as antioxidant, anti-inflammatory, and cardioprotective effects [5,6]. As a result, hawthorn is regarded as an excellent raw material for functional foods. Over the years, hawthorn has been developed into many products, including teas, jams, juices, dietary supplements, and pharmaceutical formulations [5,7]. These diverse applications underscore its importance in the food and health industries, offering substantial economic value and nutritional benefits.

One of the hawthorn’s most remarkable characteristics is its adaptability to diverse environmental conditions, enabling it to thrive in various climates and altitudes. However, the accumulation of bioactive compounds in hawthorn is significantly influenced by environmental factors such as temperature, solar radiation, and oxygen levels [8]. This variability in bioactive content directly impacts the nutritional composition and functional properties of hawthorn-derived products [9,10], posing challenges for ensuring consistent quality and standardization. For instance, higher altitudes are often characterized by elevated UV radiation and lower temperatures [11,12], which can stimulate the synthesis of phenolic compounds and antioxidants in plants [13,14]. UV radiation induces protective metabolic responses while cooler temperatures reduce oxidative stress, thereby promoting the accumulation of antioxidant compounds [15]. Similar altitude-driven effects have been observed in other fruits like apples, blueberries, and strawberries [11], suggesting that altitude plays a crucial role in shaping the nutritional profiles of numerous plant species [16,17].

Despite its adaptability, hawthorn is not impervious to environmental influences, which can complicate the classification of hawthorn products into quality grades or the refinement of their processing for specific applications. Therefore, comprehending how environmental factors, particularly altitude, impact hawthorn’s nutritional and functional quality is imperative for optimizing cultivation practices and processing strategies.

To address this challenge, this study investigates the influence of altitude on the accumulation of bioactive compounds in hawthorn. Specifically, it examines the levels of key nutrients such as vitamin C, dietary fiber, flavonoids, and phenolic acids in hawthorn cultivated at different altitudes. This study systematically analyzes altitude-related patterns in hawthorn’s nutritional composition using descriptive statistics, correlation analysis, and Principal Component Analysis (PCA). These analyses aim to uncover how altitude affects hawthorn synthesis and the accumulation of functional compounds. The findings of this study have significant implications for agriculture and industry, providing valuable insights into optimizing growing environments to enhance hawthorn yield quality and consistency in the agricultural sector. Additionally, understanding altitude-driven variations in bioactive compounds contributes to product refinement, quality grading, and targeted processing within the food and health industries. Moreover, this research deepens our understanding of how environmental factors influence functional food quality, thereby supporting sustainable cultivation and utilization of hawthorn. These insights benefit stakeholders ranging from farmers to industries and serve as a fundamental basis for further investigation into the environmental impacts on functional food resources.

## 2. Materials and Methods

### 2.1. Sample Collection

**Figure 1:**
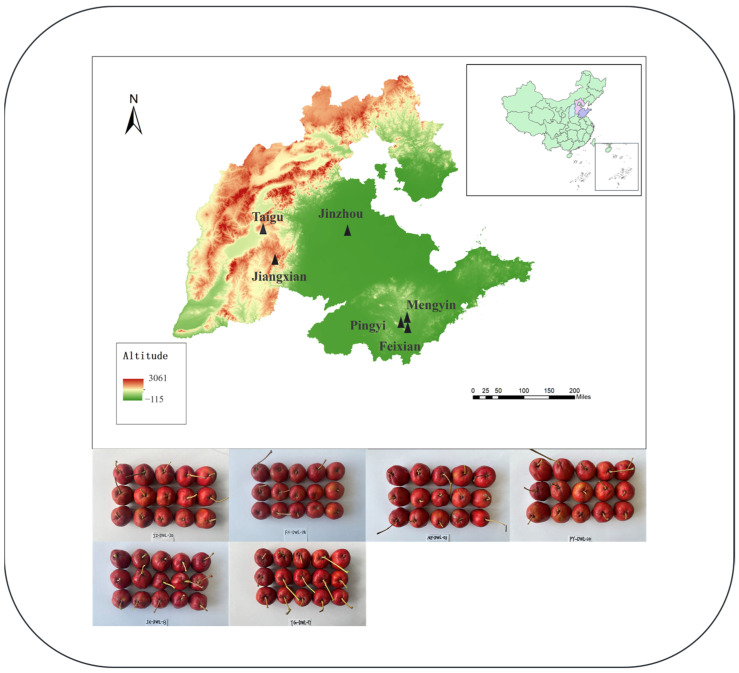
Sample sampling area and sample information diagram.

The hawthorn variety investigated was *Crataegus pinnatifida* Bunge var. *major* (“Dawuleng”). Fully mature fruits were manually harvested in October 2023 from six locations across China, encompassing diverse altitudes, as follows: JZ (Jinzhou, 47 m), FX (Feixian, 168 m), MY (Mengyin, 211 m), PY (Pingyi, 225 m), JX (Jiangxian, 842 m), and TG (Taigu, 1098 m). The sampling sites selected involved three large hawthorn growing provinces: Shandong, Hebei, and Shanxi. Subsequently, the samples were transported under controlled conditions of 4 °C to ensure their preservation and integrity for subsequent analysis. Specific sample information is presented in Table S1 and Figure 1.

### 2.2. Nutritional Composition Analysis

#### 2.2.1. Dietary Fiber

The dietary fiber content was measured using the enzymatic–gravimetric method in accordance with GB 5009.88-2014 [18]. Briefly, the samples were enzymatically hydrolyzed to remove starch and protein, followed by filtration and gravimetric determination of the residual fiber.

#### 2.2.2. Protein

The Kjeldahl method determined the protein content according to GB 5009.5-2016 [19]. Samples were digested with sulfuric acid, and the total nitrogen content was determined. The nitrogen content was then converted to protein using a conversion factor 6.25. The results were expressed as grams of protein per 100 g of fresh fruit.

#### 2.2.3. Fat

The total fat content was determined using the acid hydrolysis method described in GB 5009.6-2016 [20]. The samples were hydrolyzed with hydrochloric acid, followed by extraction with petroleum ether. After the solvent was evaporated, the fat content was weighed and expressed as grams of fat per 100 g of fresh fruit.

#### 2.2.4. Moisture

The moisture content was determined using the direct drying method described in GB 5009.3-2016 [21]. Approximately 5 g of each sample was weighed and dried at 105 °C until a constant weight was obtained. The moisture content was expressed as grams per 100 g of fresh weight (g/100 g).

#### 2.2.5. Ash

The ash content was determined according to GB 5009.4-2016 [22]. Samples were incinerated in a muffle furnace (Longkou Electric Furnace Manufacturing Factory, SX2-12-10) at 550 °C for 4 h until all organic material was combusted. The residue was weighed, the ash content was expressed as grams per 100 g of fresh weight (g/100 g).

#### 2.2.6. Soluble Solids

The soluble solid content was determined using a refractometer, following the method outlined in NY/T 2637-2014 [23]. The fruit juice was extracted, and the total soluble solids were measured at 20 °C. The results were expressed as a percentage of the total fruit weight.

### 2.3. HPLC Determination of Vitamin C (VC)

The vitamin C content was measured using high-performance liquid chromatography (HPLC) as per GB 5009.86-2016 [24]. Samples were extracted with metaphosphoric acid, filtered, and analyzed using HPLC (Agilent, Santa Clara, CA, USA, 1200) with a C18 column. The mobile phase consisted of water (0.1% formic acid) and methanol, with a 1.0 mL/min flow rate. The UV detector was set at 254 nm, and the results were expressed as milligrams of vitamin C per kilogram of fresh fruit (mg/kg).

### 2.4. LC-MS/MS Determination of Hyperoside, Quercetin, Rutin, Protocatechuic Acid, and Chlorogenic Acid

The content of hyperoside, quercetin, rutin, protocatechuic acid, and chlorogenic acid was determined using LC-MS/MS according to NY/T4307-2023 and NY/T3548-2020 [25,26]. Samples were extracted with methanol, filtered, and analyzed using LC-MS/MS (Agilent-1290-6470B) equipped with a C18 column. The mobile phase comprised acetonitrile and water (0.1% formic acid), with a flow rate of 0.3 mL/min. The mass spectrometer (Agilent-1290-6470B) was operated in MRM mode to identify and quantify the compounds. The results are expressed in micrograms per kilogram of fresh fruit.

### 2.5. Determination of Trace Elements

The trace elements were determined using inductively coupled plasma mass spectrometry (ICP-MS) and inductively coupled plasma optical emission spectrometry (ICP-OES) according to GB 5009.268-2016 [27]. Samples were digested with a mixture of nitric acid and perchloric acid using high-purity reagents. The digested solution was analyzed using ICP-MS and ICP-OES to precisely determine the trace elements. The results were expressed in milligrams per kilogram of fresh fruit.

### 2.6. Data Analysis

Data were analyzed using SPSS software (version 26.0, IBM, Armonk, NY, USA) and Microsoft Office (2021 version). Sampling maps were created using ArcMap (version 10.7). Descriptive statistics and correlation analyses were performed in SPSS, and Pearson correlation coefficients were calculated. Correlation plots were generated using Origin software (2024 version). Bar charts were created using GraphPad Prism 8.0.2. Statistical significance was set at *p* < 0.05.

## 3. Results

### 3.1. Distinct Nutritional and Bioactive Profiles of Hawthorn Differentiated by Altitude

To comprehensively assess the impact of altitude on hawthorn samples, a total of 20 nutritional indicators were analyzed, encompassing essential dietary components (vitamin C, dietary fiber, protein, fat, moisture, ash, and soluble solids), functional bioactive compounds (hypericin, quercetin, rutin, dihydrocaffeic acid, protocatechuic acid, and chlorogenic acid), as well as trace elements (potassium, calcium, iron, phosphorus, zinc, selenium, and strontium). This dataset overviews the bioactive compounds’ nutritional composition and distribution in the hawthorn samples collected from regions at varying altitudes.

**Figure 2:**
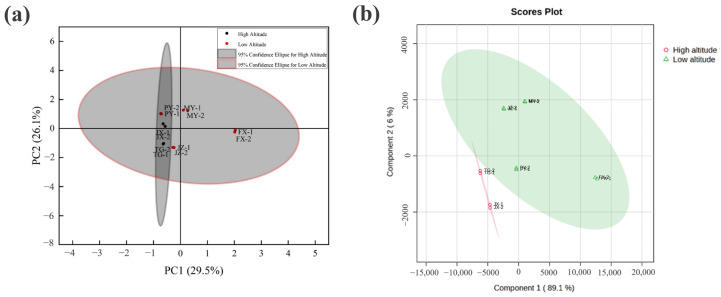
Multivariate analysis of high- and low-altitude samples: (**a**) PCA score plot and (**b**) PLS-DA score plot.

The patterns in these indicators were investigated, and the potential influence of altitude was assessed using Principal Component Analysis (PCA) and Partial Least Squares Discriminant Analysis (PLS-DA). As depicted in Figure 2a, PCA revealed that PC1 and PC2 accounted for 29.5% and 26.1% of the total variance, respectively, contributing to a combined total of 55.6%. Including the third component increased the explained variance to 75.09%, ensuring a comprehensive dataset representation. The PCA plots demonstrated natural clustering of the hawthorn samples into two groups along PC1, corresponding to low- and high-altitude regions.

Similarly, the PLS-DA results (Figure 2b) confirmed the inherent clustering observed in PCA, demonstrating a distinct sample separation based on altitude. Component 1, accounting for 89.1% of the total variance, was the principal axis distinguishing the low-altitude samples (MY, PY, JZ, FX) from the high-altitude samples (TG, JX). The samples from the low-altitude regions exhibited a tightly clustered distribution within the green confidence ellipse, reflecting high internal consistency and minimal variability. In contrast, the high-altitude samples displayed greater dispersion with certain data points falling outside of the confidence ellipse, indicating heightened heterogeneity potentially attributed to environmental adaptation.

The multivariate analyses demonstrate a significant influence of altitude on the hawthorn samples’ nutritional and bioactive profiles. The observed segregation of samples based on altitude underscores its pivotal role in modulating the synthesis and accumulation of specific compounds. This differentiation can likely be attributed to variations in environmental factors, including temperature, UV radiation, and soil composition. As revealed by PCA and PLS-DA, the natural clustering of hawthorn samples by altitude underscores the significant impact of altitude on their nutritional profiles. The distribution of individual components, including basic nutrients, bioactive compounds, and trace elements, was analyzed in detail to further elucidate these differences.

### 3.2. Basic Nutritional Components in Hawthorn Are Not Significantly Affected by Altitude

To assess the potential impact of altitude on these parameters, a comprehensive comparison was conducted to analyze the fundamental nutritional components in hawthorn, encompassing vitamin C (VC), dietary fiber, protein, fat, moisture, ash, and soluble solids. Samples collected from high-altitude and low-altitude regions were subjected to rigorous analysis while employing statistical significance tests for discerning any disparities.

**Figure 3:**
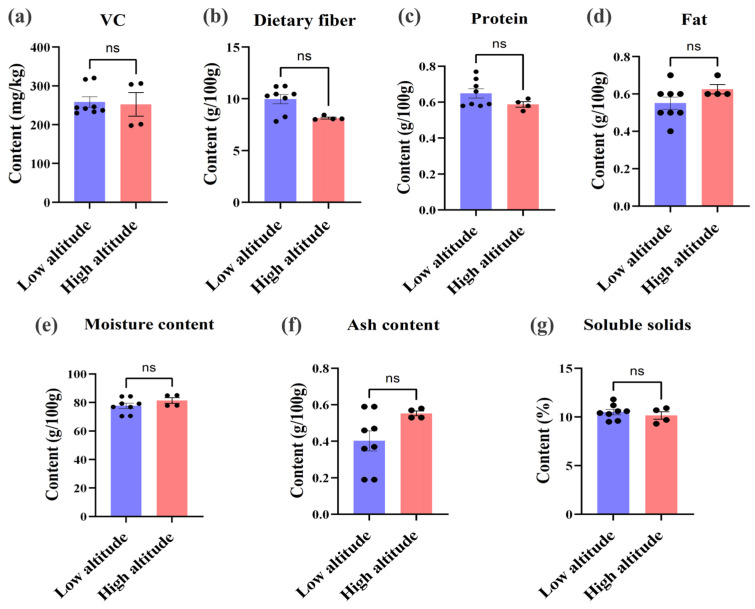
The effect of altitude on seven essential nutrients in hawthorn: (**a**) VC, (**b**) dietary fiber, (**c**) protein, (**d**) fat, (**e**) moisture content, (**f**) ash content, (**g**) soluble solids. Note: ns indicates no significant difference (*p* > 0.05).

As depicted in Figure 3, the levels of vitamin C (VC) (Figure 3a), dietary fiber (Figure 3b), protein (Figure 3c), fat (Figure 3d), moisture (Figure 3e), ash (Figure 3f), and soluble solids (Figure 3g) exhibited no statistically significant disparities between hawthorn samples from high- and low-altitude regions (*p* > 0.05 for all parameters). Although minor variations were observed, such as slightly elevated levels of VC, dietary fiber, and soluble solids in the low-altitude samples and marginally increased ash content in the high-altitude samples, none reached statistical significance. These findings suggest that altitude does not impact the accumulation of these fundamental nutritional components in hawthorn, indicating remarkable stability across varying altitudes.

The absence of significant disparities in the fundamental nutritional constituents across different altitudes implies that hawthorn cultivated in diverse regions can uphold consistent levels of these indispensable nutrients. Nevertheless, PCA disclosed the formation of two distinct clusters based on the overall nutritional composition between samples from high- and low-altitude regions. This suggests potential discrepancies in other nutritional categories, such as bioactive compounds and trace elements. To further investigate these variations, this study conducted a comprehensive comparative analysis of these functional and elemental components in subsequent sections.

### 3.3. Functional Bioactive Compounds in Hawthorn Exhibit Altitude-Dependent Variations

A comprehensive analysis was conducted on hawthorn samples to assess the levels of the following six key bioactive compounds: hypericin, quercetin, rutin, protocatechuic acid, chlorogenic acid, and dihydrocaffeic acid. These compounds possess significant functional properties and are essential indicators of hawthorn’s nutritional and medicinal value. By comparing the distribution patterns of these compounds between high- and low-altitude groups, this study aimed to evaluate the influence of altitude on their accumulation.

**Figure 4:**
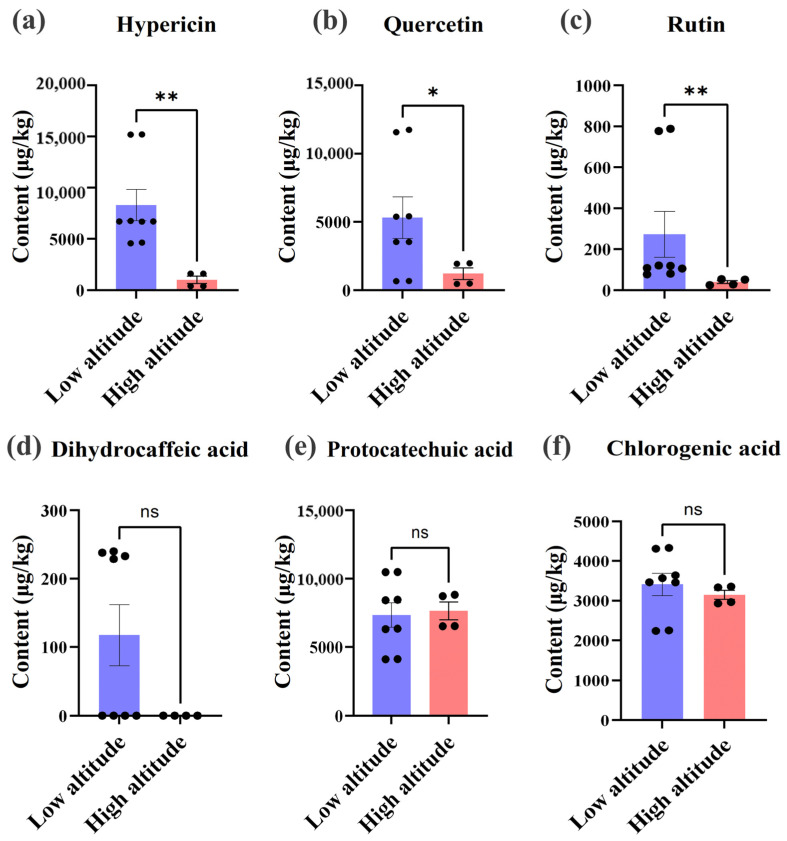
Effects of altitude on the bioactive compounds in hawthorn fruit: (**a**) hyperoside, (**b**) quercetin, (**c**) rutin, (**d**) dihydrocaffeic acid, (**e**) protocatechuic acid, and (**f**) chlorogenic acid. Note: * *p* < 0.05, ** *p* < 0.01, ns: No significant difference (*p* > 0.05).

The results revealed a significant impact of altitude on the accumulation of bioactive compounds. Notably, the low-altitude samples exhibited significantly higher levels of hypericin content compared to the high-altitude samples (*p* < 0.01) (Figure 4a). Among the low-altitude regions, the FX sample displayed the highest hypericin content (approximately 15,201 μg/kg), followed by MY (6695.88 μg/kg). In contrast, TG, representing a high-altitude sample, demonstrated the lowest level of hypericin synthesis. Similarly, the quercetin content (Figure 4b) exhibited a significant increase in the low-altitude samples compared to the high-altitude samples (*p* < 0.05). The maximum concentration of quercetin was observed in FX (11,655.92 μg/kg), followed by MY (5395.06 μg/kg), while TG displayed the lowest value (466.46 μg/kg). The rutin levels (Figure 4c) exhibited a significant increase in the low-altitude samples (*p* < 0.01). FX displayed the highest rutin content (782.54 μg/kg), while the other low-altitude samples, namely MY and PY, showed moderate levels ranging from 100 to 120 μg/kg. In the high-altitude regions, there was a sharp decline in the rutin content, with JX exhibiting the lowest level (26 μg/kg). These results underscore the importance of altitude-specific environmental factors in shaping the bioactive profiles of hawthorn, offering valuable insights for optimizing cultivation strategies to enhance bioactive compound accumulation.

In contrast, the contents of protocatechuic acid (Figure 4d), chlorogenic acid (Figure 4e), and dihydrocaffeic acid (Figure 4f) showed no significant differences between the low- and high-altitude groups (*p* > 0.05), suggesting that altitude-related environmental factors influence these compounds less. Their stability across different altitudes may be attributed to their essential roles in fundamental plant metabolic processes, which remain largely unaffected by varying ecological conditions. This stability highlights the robust nature of these compounds in maintaining their levels regardless of environmental changes. The results revealed that hypericin, quercetin, and rutin contents were significantly higher in the low-altitude samples, suggesting their sensitivity to environmental factors such as light intensity, temperature, and UV radiation. Conversely, protocatechuic acid, chlorogenic acid, and dihydrocaffeic acid exhibited no significant differences across different altitudes, reflecting their higher environmental adaptability. These findings highlight the diverse strategies employed by hawthorn to adapt to varying environmental conditions and emphasize the influence of altitude on the accumulation of specific functional bioactive compounds.

### 3.4. Altitude-Dependent Variations in Mineral Element Accumulation in Hawthorn

This study investigated the accumulation patterns of essential mineral elements in hawthorn samples collected from diverse altitudes, aiming to assess the influence of altitude on their concentrations. Our analysis unveiled significant variations associated with altitude for specific elements, while others exhibited consistent levels across different altitudes.

**Figure 5:**
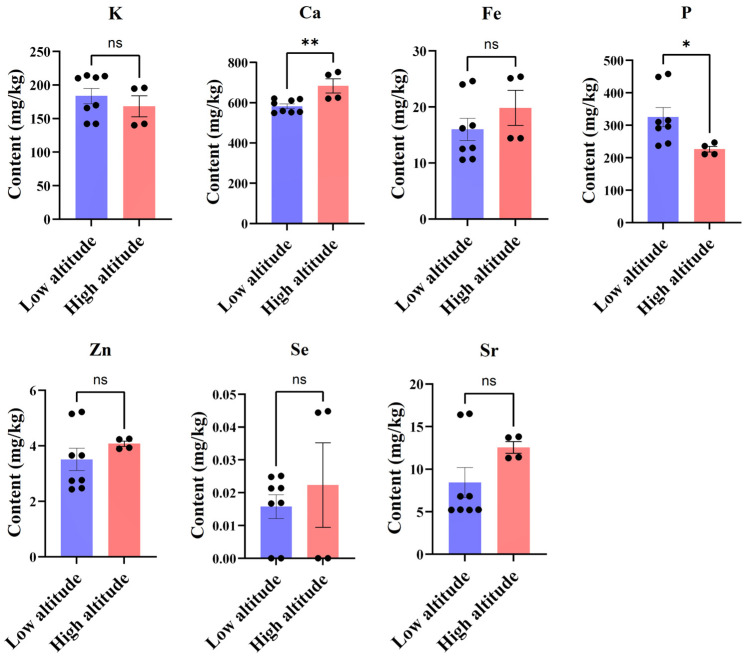
Descriptive analysis of seven trace elements in hawthorn at different altitudes. Note: * *p* < 0.05, ** *p* < 0.01, ns: No significant difference (*p* > 0.05).

The calcium content was significantly higher in the high-altitude samples than the low-altitude samples (*p* < 0.01) (Figure 5). This statement is valid especially for the TG samples (1098 m altitude). Calcium tends to accumulate in plant tissues under the low temperatures typical of high-altitude environments. Lower temperatures at high altitude slow plant growth and metabolism, which promotes the long-term accumulation of calcium. This may be one of the factors in this outcome indicating that elevated conditions at higher altitudes promote calcium accumulation, potentially due to environmental factors such as soil composition, enhanced sunlight intensity, or lower temperatures. These factors may stimulate calcium uptake or increase calcium reserves to enhance cell wall stability, facilitating plant adaptation to colder environments. In contrast, the phosphorus content was significantly lower in the high-altitude samples than in the low-altitude samples (*p* < 0.05). This reduction in phosphorus accumulation at higher altitudes could be attributed to environmental constraints such as temperature or light intensity, which may suppress phosphorus absorption.

No significant differences were observed in the levels of potassium, iron, zinc, selenium, and strontium among altitude groups (*p* > 0.05 for all) (Figure 5). Potassium showed consistent accumulation across different altitudes due to its stable absorption mechanisms as an essential macronutrient for plant growth. Iron content remained uniform because of its constant requirement in plant metabolic processes and availability in soil. Zinc also exhibited no altitude-related variation despite being a crucial micronutrient involved in enzymatic functions and growth, indicating minimal influence from environmental changes on its absorption. Selenium content demonstrated stability across different altitudes, primarily derived from soil with lower dependence on environmental variations. Strontium levels remained consistent across different altitudes (Table S4), with accumulation being influenced more by soil mineral composition than altitude-related environmental factors.

The results demonstrate significant altitude-related differences in calcium and phosphorus, while the other tested elements exhibit relatively stable levels across different altitudes. These findings suggest that altitude can influence the accumulation of specific nutrients, particularly those involved in structural or adaptive functions such as calcium and phosphorus. In contrast, others maintain consistent levels due to robust absorption and metabolic regulation mechanisms. These results offer valuable insights into the impact of altitude on mineral nutrient distribution in hawthorn plants and highlight their adaptive strategies under varying environmental conditions.

### 3.5. Altitude Shapes Nutritional Profiles and Reveals Metabolic Interactions in Hawthorn

**Figure 6:**
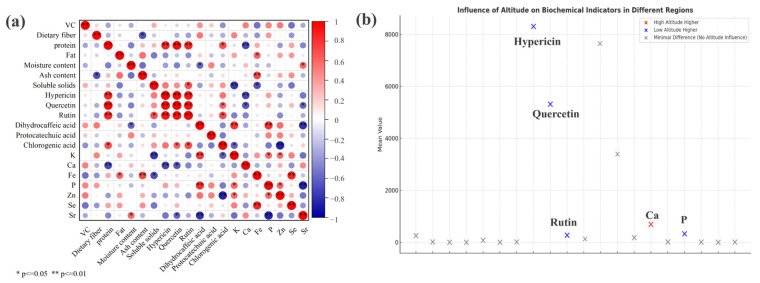
(**a**): Heat map of the correlation between variables (red indicates a positive correlation; Blue indicates a negative correlation; * *p* ≤ 0.05, ** *p* ≤ 0.01); (**b**): The distribution of altitude to the content of each index.

To investigate the relationships among nutritional indicators and elucidate the mechanisms underlying their altitude-dependent distribution, a correlation analysis was conducted on 20 components, including basic nutrients, bioactive compounds, and mineral indices in the hawthorn samples (Figure 6a). This analysis revealed complex interactions among these components, providing insights into their metabolic connections and accumulation characteristics.

Protein levels exhibited significant positive correlations with hyperoside (r = 0.857, *p* < 0.01) and rutin (r = 0.849, *p* < 0.01), as well as a highly significant positive correlation with quercetin (r = 0.947, *p* < 0.01). These correlations suggest interconnected biosynthetic pathways, where higher protein content may facilitate the synthesis of certain bioactive compounds. Similarly, hyperoside and quercetin displayed a highly significant positive correlation (r = 0.944, *p* < 0.01), further supporting the hypothesis of shared or co-regulated metabolic pathways. Chlorogenic acid was positively correlated with both rutin (r = 0.701, *p* < 0.05) and protein (r = 0.699, *p* < 0.05), indicating coordinated metabolic roles. Dihydrocaffeic acid exhibited a significant negative correlation with moisture content (r = −0.594, *p* < 0.05), suggesting that it stabilizes better under drier conditions, which may explain its consistent levels across different altitudes. Ash content demonstrated a significant negative correlation with dietary fiber (r = −0.669, *p* < 0.05), reflecting a potential trade-off between mineral content and structural carbohydrates.

Trace elements showed similarly intricate relationships with both bioactive compounds and basic nutrients. Calcium exhibited highly significant negative correlations with protein (r = −0.713, *p* < 0.01) and hyperoside (r = −0.716, *p* < 0.01), as well as a significant negative correlation with quercetin (r = −0.660, *p* < 0.05). These findings suggest competitive or regulatory interactions in resource allocation, consistent with the higher calcium levels and reduced protein and hyperoside concentrations observed in the high-altitude samples. Potassium showed a highly significant negative correlation with soluble solids (r = −0.772, *p* < 0.01) and a significant negative correlation with chlorogenic acid (r = −0.612, *p* < 0.05), but a highly significant positive correlation with dihydrocaffeic acid (r = 0.796, *p* < 0.01), reflecting its complex roles in regulating soluble and bioactive compounds. Phosphorus showed a highly significant positive correlation with dihydrocaffeic acid (r = 0.808, *p* < 0.01) and a significant positive correlation with potassium (r = 0.597, *p* < 0.05), suggesting coordinated roles in metabolic pathways. Zinc demonstrated a significant negative correlation with chlorogenic acid (r = −0.846, *p* < 0.01) but positive correlations with both potassium (r = 0.577, *p* < 0.05) and phosphorus (r = 0.587, *p* < 0.05), underscoring its interaction with trace elements and bioactive compounds. Selenium exhibited a significant positive correlation with iron (r = 0.809, *p* < 0.01), while strontium was significantly positively correlated with moisture (r = 0.588, *p* < 0.05), significantly negatively correlated with quercetin (r = −0.580, *p* < 0.05), and highly significantly negatively correlated with dihydrocaffeic acid (r = −0.792, *p* < 0.01) and phosphorus (r = −0.856, *p* < 0.01). These findings highlight strontium’s role in balancing mineral content and bioactive compound regulation.

Overall, the distribution of nutritional indicators exhibited significant variations between high- and low-altitude samples (Figure 6b). Calcium content was notably elevated at higher altitudes, reflecting the influence of environmental factors such as reduced oxygen levels and climatic stress. Conversely, hypericin, quercetin, rutin, and phosphorus were found to be significantly more abundant at lower altitudes, likely driven by disparities in temperature, humidity, and other environmental conditions. Potassium, iron, zinc selenium, and strontium did not display significant differences between altitudes, thus indicating their stability and minimal susceptibility to environmental fluctuations. These findings comprehensively summarize altitude-dependent distribution patterns and correlations among all analyzed indicators.

## 4. Discussion

This study comprehensively evaluates altitude-dependent variations in hawthorn’s nutritional, bioactive, and mineral profiles. The findings underscore the stability of essential nutritional components while highlighting the significant impact of altitude on specific bioactive compounds and minerals. Moreover, they elucidate the intricate metabolic interactions among these indicators. These insights deepen our understanding of hawthorn’s adaptive strategies and provide valuable guidance for its cultivation and utilization in functional food production.

### 4.1. Stability of Basic Nutritional Components Across Different Altitudes

The consistent presence of fundamental nutritional components, such as dietary fiber, protein, fat, moisture, ash, and soluble solids across varying altitudes, highlights the robust metabolic processes of hawthorn. These essential constituents form the basis of hawthorn’s nutritional value and remain unaffected by environmental variations, ensuring a stable dietary quality. This stability offers significant advantages to the food industry as it guarantees the reliability of hawthorn-derived products regardless of cultivation altitude. Similar findings in other crops suggest that primary metabolic processes are evolutionarily conserved, providing resilience against environmental fluctuations [28,29,30].

However, the clear clustering of samples in PCA and PLS-DA indicates compositional differences between high- and low-altitude groups, pointing to variations in secondary metabolites and trace elements. These differences align with previous studies on altitude’s impact on fruit crops, such as apples and grapes, where environmental stressors influenced secondary metabolic pathways without affecting primary metabolites. For example, research on apples found that phenolic compounds varied with altitude, while sugars and organic acids remained constant [31]. Similarly, a study on Ginkgo biloba leaves revealed that the secondary metabolic pathways responsible for flavonoid biosynthesis are significantly influenced by altitude, while primary metabolic processes remain largely unaffected [32]. This highlights the selective impact of environmental factors on secondary metabolite production, with minimal effects on other metabolic activities. Further investigation into these secondary metabolites and their environmental adaptability is warranted to optimize hawthorn cultivation.

### 4.2. Altitude-Dependent Variations in Bioactive Compounds and Mineral Elements

The significant altitude-dependent accumulation of hypericin, quercetin, and rutin in low-altitude samples demonstrates their sensitivity to environmental factors such as temperature, light intensity, and UV radiation. Previous studies on medicinal plants, including *Ginkgo biloba* and *Camellia sinensis*, similarly reported enhanced flavonoid and phenolic compound accumulation in regions with moderate climatic conditions. For instance, *Ginkgo biloba* leaves collected from lower altitudes exhibited higher flavonoid content, attributed to favorable growing conditions [33]. In *Camellia sinensis*, tea plants grown at lower elevations had increased catechin concentrations, linked to optimal temperature and light conditions [34]. These findings suggest that low-altitude regions provide favorable conditions for the biosynthesis of secondary metabolites due to reduced environmental stress and increased photosynthetic activity.

In contrast, the stability of protocatechuic acid, chlorogenic acid, and dihydrocaffeic acid across different altitudes reflects their fundamental roles in primary metabolism. Studies on *Arabidopsis thaliana* and rice have shown that core metabolites associated with growth and development are less responsive to environmental changes, ensuring homeostasis. For example, in *Arabidopsis thaliana*, primary metabolite levels remained stable under varying environmental conditions, indicating a robust metabolic framework [35]. Similarly, rice plants maintained consistent levels of essential amino acids across different growing environments, highlighting metabolic stability [36]. This stability highlights the importance of these compounds in maintaining hawthorn’s baseline metabolic processes across varying ecological conditions. Our results showed that hypericin, quercetin, and rutin levels were significantly elevated in low-altitude regions, whereas dihydrocaffeic acid, protocatechuic acid, and chlorogenic acid exhibited no significant variation across different altitudes. This disparity can be attributed to the distinct roles of these compounds in plant metabolism and their differential sensitivity to environmental factors. Hypericin, quercetin, and rutin are typical secondary metabolites that play crucial roles in plant defense mechanisms. Their biosynthesis is highly responsive to external factors such as temperature, humidity, and precipitation. For example, our study revealed that low-altitude regions experienced higher temperatures (particularly from May to October) (Figure S1), increased humidity (especially in July and August), and more substantial precipitation (notably in July and September) compared to high-altitude regions [13,37]. These environmental conditions likely facilitated the accumulation of these compounds in low-altitude regions. In contrast, dihydrocaffeic acid, protocatechuic acid, and chlorogenic acid, while also classified as secondary metabolites, are phenolic acids that play relatively stable roles within the phenylpropanoid pathway. Their biosynthesis exhibits lower sensitivity to environmental changes, which may account for the lack of significant differences in their levels across different altitudes [38,39].

Distinct altitude-dependent variations were observed in the mineral element patterns of hawthorn. This study emphasizes significant altitude-related differences in hawthorn’s mineral content, particularly elevated calcium levels in samples collected from high-altitude regions. Calcium plays a crucial role in plant adaptation to environmental stress, acting as a structural component of cell walls and a signaling molecule in stress responses. Plants are exposed to low temperatures, increased UV radiation, and substantial diurnal temperature variations at high altitudes. These stressors necessitate enhanced structural integrity and stress signaling. Calcium accumulation strengthens cell walls, improving mechanical resilience and cold tolerance, critical for survival in harsh alpine environments. Additionally, calcium’s role in intracellular signaling facilitates rapid responses to environmental challenges, further supporting its accumulation in high-altitude hawthorn [40]. In contrast, phosphorus content was significantly lower in high-altitude hawthorn samples, a pattern commonly associated with reduced phosphorus availability and uptake efficiency under cooler soil conditions. Studies on high-altitude vegetation have consistently reported similar trends, with phosphorus limitations being attributed to reduced soil phosphorus solubility and slower root metabolic activity in cold environments. For example, research on *Quercus aquifolioides* in the Hengduan Mountains demonstrated a significant decrease in phosphorus content at higher elevations, reflecting the challenges of nutrient acquisition in alpine ecosystems [41]. The observed lower phosphorus levels in high-altitude hawthorn may represent an adaptive strategy prioritizing the allocation of resources to other essential processes.

This analysis revealed that, among the seven detected mineral elements (potassium, calcium, iron, phosphorus, strontium, zinc, and selenium), only calcium and phosphorus exhibited significant differences between high-altitude and low-altitude regions. Calcium concentrations were significantly higher in high-altitude areas, whereas phosphorus levels were significantly elevated in low-altitude areas. This disparity can be attributed to the distinct physiological roles of these minerals and their sensitivity to environmental conditions. Calcium, as a key structural component of plant cell walls, tends to accumulate in plant tissues under cooler conditions typical of high-altitude environments. The lower temperatures at higher altitudes can slow down plant growth and metabolism, facilitating calcium accumulation over time [42]. Conversely, phosphorus plays a critical role in energy transfer and metabolic processes, and its higher levels in low-altitude regions may be due to the warmer temperatures, higher humidity, and increased precipitation observed at lower altitudes. These conditions likely enhance soil nutrient availability and phosphorus uptake by plants, resulting in elevated phosphorus levels in low-altitude regions [43].

However, it is worth noting that when comparing specific sampling regions within the same altitude category, such as JZ (47 m, low altitude) and JX (842 m, high altitude), the phosphorus contents showed no significant difference (240.50 ± 3.50 μg/g vs. 241.50 ± 5.50 μg/g). This indicates that, while significant differences in phosphorus levels are observed at the broader altitude group level, localized factors such as microclimates, soil composition, or regional plant physiology may also play a role in influencing mineral content.

Interestingly, the levels of other mineral elements, including potassium, iron, zinc, selenium, and strontium, remained stable across different altitudes. This suggests that the concentrations of these elements may be less susceptible to environmental variations and more strongly influenced by the inherent soil mineral composition, which was not examined in this study. These minerals play crucial roles in fundamental metabolic processes, and their stability implies robust regulatory mechanisms, ensuring essential nutrient availability irrespective of environmental variations. Such resilience aligns with findings in lentils, where mineral profiles (including potassium, iron, and zinc) remained consistent across different harvesting times and environmental conditions [44]. This underscores hawthorn’s capacity to adapt to diverse growth environments without compromising its nutritional quality.

### 4.3. Implications for Cultivation, Utilization, and Future Research

Our findings significantly affect hawthorn cultivation and its use in functional food production. Low-altitude regions, characterized by higher concentrations of bioactive compounds such as quercetin, hypericin, and rutin, are ideal for producing antioxidant-rich hawthorn products. These antioxidant compounds are associated with numerous health benefits, including reduced oxidative stress and enhanced cardiovascular health, making low-altitude hawthorn cultivation valuable for the nutraceutical and health food industries. Conversely, the high-altitude cultivation of hawthorn may enhance calcium-enriched varieties, catering to dietary needs associated with bone health and structural support, as higher calcium content was observed in these regions. Similar altitude-based cultivation strategies have been successfully implemented in medicinal plants such as *Rhodiola rosea* and *Panax notoginseng*, where targeted altitude optimization enhanced the concentration of active ingredients [45,46].

Beyond cultivation, altitude-specific differences in hawthorn composition also inform tailored processing strategies to maximize its functional benefits. Low-altitude hawthorn, with higher concentrations of flavonoids and phenolics, is well-suited for processing into teas, extracts, and supplements that target antioxidant properties. High-altitude hawthorn, enriched in calcium, may be processed into products such as calcium-fortified powders, beverages, or snacks aimed at promoting bone health. Processing techniques, such as freeze-drying and vacuum concentration, should be optimized to preserve these altitude-dependent bioactive and mineral profiles, ensuring that the end products retain their nutritional and functional integrity.

Future research should explore integrating advanced omics technologies to elucidate further the genetic and molecular mechanisms governing these altitude-dependent variations. Transcriptomics and metabolomics can provide insights into the regulatory networks influencing the synthesis and accumulation of bioactive compounds and minerals. Moreover, investigating the soil microbiome and its interactions with hawthorn roots across different altitudes could unveil critical environmental factors that drive these metabolic adaptations. Clinical studies evaluating the health effects of hawthorn-derived bioactive compounds are also warranted. These studies could focus on validating the therapeutic potential of hawthorn products rich in specific bioactive compounds such as quercetin and hypericin, paving the way for developing targeted nutraceuticals. Additionally, modeling studies that predict the effects of climate change on hawthorn’s metabolic profiles across different altitudes would be invaluable for future agricultural planning.

## 5. Conclusions

This study comprehensively examined the impact of altitude on hawthorn’s nutritional, bioactive, and mineral profiles, revealing significant altitude-dependent clustering and variations. While fundamental nutritional components such as dietary fiber, protein, fat, moisture, ash, and soluble solids remained stable across different altitudes, highlighting robust metabolic processes that ensure dietary quality, altitude had a marked influence on bioactive compounds. Low-altitude samples demonstrated significantly higher levels of hypericin, quercetin, and rutin, suggesting that moderate environmental conditions with optimal light intensity, temperature, and precipitation favor their biosynthesis. In contrast, high-altitude samples exhibited elevated calcium content, aligning with its role in supporting cold stress adaptation and structural integrity, while phosphorus levels were higher at low altitudes due to enhanced soil availability and uptake efficiency in warmer and wetter conditions. Interestingly, while significant differences were observed in phosphorus levels between the broader altitude groups, specific regions such as JZ (low altitude) and JX (high altitude) displayed no significant differences, highlighting the potential influence of localized factors such as microclimates or site-specific physiology. Other mineral elements, including potassium, iron, zinc, selenium, and strontium, remained stable across different altitudes, reflecting their fundamental roles in metabolic regulation and their resilience to environmental fluctuations. Correlation analysis revealed metabolic interconnections, such as positive associations between protein and flavonoids, suggesting shared biosynthetic pathways, while the stable distribution of minerals underscored robust regulatory mechanisms. These findings illustrate hawthorn’s adaptive strategies, maintaining essential nutrient stability while exhibiting flexibility in secondary metabolism. This research emphasizes the importance of altitude-specific cultivation strategies and provides valuable insights into leveraging environmental influences to optimize hawthorn’s potential as a sustainable resource for health and nutrition applications.

## Data Availability

The original contributions presented in this study are available in the article/Supplementary Materials.

## Supplementary Materials

The following supporting information can be downloaded at: https://www.mdpi.com/article/10.3390/foods14020241/s1, Figure S1: Environmental data analysis of each sampling site. (a) Humidity (%); (b) Temperature (°C); (c) Precipitation (mm); (d) Surface wind speed (m/s). Red bars represent high-altitude regions and blue bars represent low-altitude regions. Data points indicate the daily mean values obtained from measurements.; Table S1: Sample information of hawthorn; Table S2: Descriptive statistics of nutrients in hawthorn with six altitude gradients; Table S3: Correlation analysis among detection indicators; Table S4: Factor analysis principal component eigenvalue and variance contribution rate. The referenced standard documents are attached at the end; Table S5: Climatological information for JZ; Table S6: Climatological information for FX; Table S7: Climatological information for MY; Table S8: Climatological information for PY; Table S9: Climatological information for JX; Table S10: Climatological information for TG [47].
