# Supplementary material for: Altitude-Driven Variations in Nutritional, Bioactive, and Mineral Profiles of Hawthorn (Crataegus spp.)
Source: Foods. 2025 Jan 14;14(2):241. doi: 10.3390/foods14020241 (PMC11765299; doi:10.3390/foods14020241)
Supplement: Supplementary file 1 [file foods-14-00241-s001.zip › foods-3371011-supplementary/final suppl/foods-3371011-supplementary.pdf]

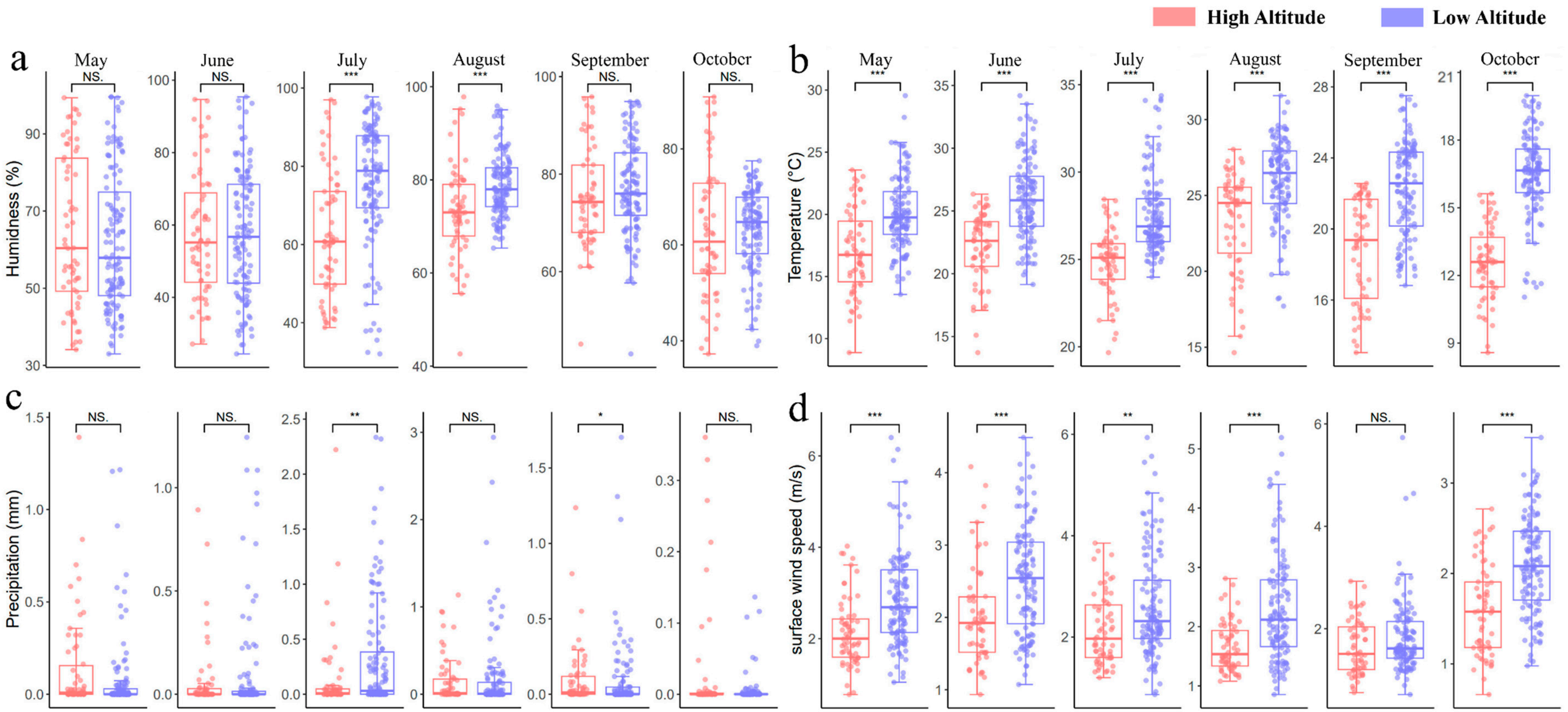

Figure S1. Environmental data analysis of each sampling site. (a) Humidity (%); (b) Temperature (°C); (c) Precipitation (mm); (d) Surface wind speed (m/s). Red bars represent high-altitude regions, and blue bars represent low-altitude regions. Data points indicate the daily mean values obtained from measurements. Detailed data information is provided in the supplementary materials.

Table S1. Sample information of hawthorn

Sample name	Altitude (m)	Elevation grouping	Sample variety	Regions	Sampling date	Longitude(°)	Latitude(°)
JZ	47	Low Altitude	‘Da Wuleng’	Hebei	2023.10.17	115.06999	37.868484
FX	168	Low Altitude	‘Da Wuleng’	Shandong	2023.10.12	118.01839	35.418678
MY	211	Low Altitude	‘Da Wuleng’	Shandong	2023.10.23	118.22711	35.8973
PY	225	Low Altitude	‘Da Wuleng’	Shandong	2023.10.19	117.78231	35.321143
JX	842	High Altitude	‘Da Wuleng’	Shanxi	2023.10.06	111.64772	35.546899
TG	1098	High Altitude	‘Da Wuleng’	Shanxi	2023.10.10	112.53386	37.332921

Note: The sampling areas are abbreviated as follows: JZ-Jinzhou; FX-Feixian; MY-Mengyin; PY-Pingyi; JX-Jiangxian; TG-Taigu.

Table S2. Descriptive statistics of nutrients in Hawthorn with six altitude gradients

Detection index	unit	Low altitude group				High altitude group	
		JZ	FX	MY	PY	JX	TG
VC	mg/kg	231.36±1.64^{a}	239.09±2.41^{d}	245.07±1.17^{c}	318.51±1.80^{a}	199.50±1.81^{f}	304.96±1.40^{b}
Dietary fiber	g/100g	11.21±0.02^{a}	8.04±0.22^{c}	10.18±0.11^{b}	10.46±0.00^{b}	8.04±0.03^{c}	8.24±0.19^{c}
protein	g/100g	0.58±0.00^{cd}	0.75±0.02^{a}	0.68±0.02^{b}	0.59±0.00^{cd}	0.61±0.01^{c}	0.57±0.02^{d}
Fat	g/100g	0.50±0.00NS	0.50±0.10NS	0.65±0.05NS	0.55±0.05NS	0.65±0.05NS	0.60±0.00NS
Moisture content	g/100g	84.25±0.05^{b}	76.85±0.05^{e}	70.45±0.05^{f}	79.15±0.05^{c}	84.95±0.05^{a}	77.85±0.05^{d}
Ash content	g/100g	0.19±0.00^{f}	0.47±0.01^{d}	0.59±0.00^{a}	0.37±0.01^{e}	0.53±0.00^{c}	0.58±0.01^{b}
Soluble solids	%	10.50±0.10^{b}	11.50±0.30^{a}	9.55±0.05^{c}	10.45±0.15^{b}	9.50±0.20^{c}	10.80±0.10^{b}
Hypericin	µg/kg	4606.29±28.65^{c}	15201.57±5.16^{a}	6695.88±8.77^{b}	6731.25±24.09^{b}	1613.34±2.35^{d}	385.89±1.46^{c}
Quercetin	µg/kg	661.08±11.01^{e}	11656.92±84.18^{a}	5395.06±7.73^{b}	3532.60±4.51^{c}	1949.63±24.57^{d}	466.46±9.63^{f}
Rutin	µg/kg	78.73±1.67^{d}	782.54±5.59^{a}	119.56±1.04^{b}	106.86±1.40^{c}	26.38±1.43^{f}	52.69±1.46^{e}
Dihydrocaffeic acid	µg/kg	0.00±0.00NS	0.00±0.00NS	233.27±4.57^{a}	236.46±3.55^{a}	0.00±0.00NS	0.00±0.00NS
Protocatechuic acid	µg/kg	6324.42±21.90^{c}	8437.92±14.22^{c}	4113.41±2.11^{f}	10474.05±7.99^{a}	8767.48±46.54^{b}	6526.71±10.08^{d}
Chlorogenic acid	µg/kg	3461.73±2.10^{c}	4321.88±9.83^{a}	3604.48±35.96^{b}	2246.10±6.34^{f}	2947.82±16.87^{e}	3346.81±10.07^{d}
K	mg/kg	167.89±2.06^{c}	142.14±0.00^{d}	213.73±0.52^{a}	210.64±0.52^{a}	195.19±0.52^{b}	141.11±1.03^{d}
Ca	mg/kg	604.00±7.00^{c}	556.50±2.50^{d}	550.50±1.50^{d}	619.50±1.50^{b}	622.50±2.50^{b}	745.00±7.00^{a}
Fe	mg/kg	10.65±0.05^{f}	16.45±0.25^{c}	24.30±0.30^{b}	12.60±0.10^{e}	25.25±0.15^{a}	14.40±0.00^{d}
P	mg/kg	240.50±3.50^{d}	293.50±2.50^{c}	312.50±2.50^{b}	453.50±4.50^{a}	241.50±5.50^{d}	211.00±0.00^{e}
Zn	mg/kg	2.45±0.02^{f}	2.75±0.01^{e}	3.65±0.00^{d}	5.19±0.04^{d}	4.24±0.10^{b}	3.91±0.02^{c}
Se	mg/kg	0.00±0.00^{c}	0.02±0.00^{b}	0.03±0.01^{b}	0.02±0.00^{b}	0.04±0.00^{a}	0.00±0.00^{c}
Sr	mg/kg	16.45±0.05^{a}	6.82±0.01^{d}	5.22±0.02^{e}	2.58±0.00^{f}	11.35±0.05^{c}	13.75±0.05^{b}

Note: Data are presented as mean ± standard error. Different letters (a-f) indicate significant differences between groups ($p < 0.05$), while the same letters indicate no significant difference. NS indicates no significant difference

Table S3.20 Correlation analysis among detection indicators

	VC	Dietary fiber	protein	Fat	Moisture content	Ash content	Soluble solids	Hypericin	Quercetin	Rutin	Dihydrocaffeic acid	Protocatechuic acid	Chlorogenicacid	K	Ca	Fe	P	Zn	Se	Sr
VC	1																			
Dietary fiber	0.171	1																		
protein	-0.356	-0.299	1																	
Fat	-0.118	-0.162	-0.069	1																
Moisture content	-0.312	-0.022	-0.498	-0.191	1															
Ash content	0.026	-.669*	0.265	0.567	-0.558	1														
Soluble solids	0.337	-0.201	0.283	-0.511	-0.019	-0.260	1													
Hypericin	-0.091	-0.035	.857**	-0.427	-0.376	-0.128	0.538	1												
Quercetin	-0.154	-0.301	.947**	-0.230	-0.483	0.191	0.443	.944**	1											
Rutin	-0.138	-0.373	.849**	-0.421	-0.269	0.021	.690*	.916**	.929**	1										
Dihydrocaffeicacid	0.433	0.522	0.040	0.211	-.594*	0.123	-0.375	0.124	0.095	-0.217	1									
Protocatechuic acid	0.228	-0.219	-0.077	-0.234	0.500	-0.249	0.272	0.169	0.123	0.186	-0.043	1								
Chlorogenic acid	-0.430	-0.316	.699*	-0.217	-0.339	0.156	0.428	0.544	.601*	.701*	-0.448	-0.500	1							
K	-0.037	0.482	-0.121	0.379	-0.151	0.043	-.772**	-0.157	-0.161	-0.485	.796**	0.033	-.612*	1						
Ca	0.506	-0.290	-.713**	0.116	0.275	0.174	0.120	-.716**	-.660*	-0.473	-0.342	0.093	-0.339	-0.397	1					
Fe	-0.557	-0.412	0.345	.608*	-0.263	.720**	-.674*	-0.098	0.167	-0.086	0.143	-0.259	0.104	0.435	-0.309	1				
P	0.499	0.407	0.112	-0.123	-0.271	-0.173	-0.006	0.388	0.285	0.088	.808**	0.497	-0.517	.597*	-0.359	-0.163	1			

Zn	0.522	-0.058	-0.381	0.379	-0.034	0.323	-0.366	-0.361	-0.271	-0.455	0.561	0.503	-.846**	.577*	0.313	0.178	.587*	1		
Se	-0.468	-0.338	0.387	0.371	-0.017	0.423	-0.535	0.132	0.313	0.062	0.245	0.289	-0.170	0.574	-0.463	.809**	0.258	0.402	1	
Sr	-0.332	-0.050	-0.466	-0.091	.588*	-0.288	0.050	-0.530	-.580*	-0.305	-.792**	-0.310	0.212	-0.544	0.474	-0.238	-.856**	-0.534	-0.505	1

*. At level 0.05 (two-tailed), the correlation was significant.

**. At level 0.01 (two-tailed), the correlation was significant.

Table S4. Factor analysis principal component eigenvalue and variance contribution rate.

Principal component	eigenvalue	Variance contribution rate (%)	Cumulative variance contribution rate (%)
1	5.898	29.490	29.490
2	5.218	26.088	55.578
3	3.903	19.514	75.093

Table S5. Climatological Information for JZ

Table S6. Climatological Information for FX

Table S7. Climatological Information for MY

Table S8. Climatological Information for PY

Table S9. Climatological Information for JX

Table S10.Climatological Information for TG

The tables S5-S10 present climate data by region [1], see annexed Excel sheet.

1 Hersbach, H., Bell, B., Berrisford, P., Biavati, G., Horányi, A., Muñoz Sabater, J., Nicolas, J., Peubey, C., Radu, R., Rozum, I., Schepers, D., Simmons, A., Soci, C., Dee, D., Thépaut, J-N. (2018): ERA5 hourly data on single levels from 1959 to present. Copernicus Climate Change Service (C3S) Climate Data Store (CDS). (Accessed on < 23-1-2024 > through www.xihe-energy.com), 10.24381/cds.adbb2d47

GB 5009.88-2014 National Standard for Food Safety Determination of Dietary Fiber in Foods

GB5009.88-2014.pdf

GB 5009.5-2016 National Standard for Food Safety Determination of Protein in Food

GB5009.5-2016.pdf

GB 5009.6-2016 National Standard for Food Safety Determination of Fat in Food

GB5009.6-2016.pdf

GB 5009.3-2016 National Standard for Food Safety Determination of Moisture in Food

GB5009.3-2016.pdf

GB 5009.4-2016 National Standard for Food Safety Determination of Ash in Foods

GB5009.4-2016.pdf

NY/T 2637-2014 Determination of soluble solids in fruits and vegetables Refractometer method

NYT2637-2014.pdf

GB 5009.86-2016 National Standard for Food Safety Determination of Ascorbic Acid in Foods

GB5009.86-2016.pdf

NY/T 4307-2023. Determination of flavonoids in Pueraria Mirifica by high performance liquid chromatography-tandem mass spectrometry

NYT4307-2023dz.pdf

NY/T 3548-2020. Determination of flavonols in fruits by liquid chromatography-mass spectrometry

NYT3548-2020.pdf

GB 5009.268-2016 National Standard for Food Safety Determination of Multi-Elements in Food

GB5009.268-2016.pdf
